# Diabetic Ketoacidosis Complicated by Thrombotic Thrombocytopenic Purpura: A Rare Association

**DOI:** 10.7759/cureus.37983

**Published:** 2023-04-22

**Authors:** Chinanu I Nwankwo, Kemar A Samuels, Akata Abung, Adetola F Oshikoya, Danish Waqar, Adekunle E Omole

**Affiliations:** 1 Medicine and Surgery, College of Medicine, Enugu State University of Science and Technology, Enugu, NGA; 2 Internal Medicine, Escuela Latinoamericana de Medicina, kingston, JAM; 3 Internal Medicine, Worcestershire Acute Hospitals NHS Trust, Worcester, GBR; 4 Internal Medicine, College of Medicine, University of Calabar, Calabar, NGA; 5 Medical School, Near East University, Nicosia, CYP; 6 General Practice, General Hospital Odan, Lagos Island, Lagos, NGA; 7 Internal Medicine/Nephrology, Loyola University Medical Center, Chicago, USA; 8 Anatomical Sciences, College of Medicine, American University of Antigua, Saint John, ATG

**Keywords:** complications of dka, thrombotic thrombocytopenic purpura, acquired ttp, diabetic keto acidosis, dka

## Abstract

Thrombotic thrombocytopenic purpura (TTP) is a rare and potentially life-threatening blood disorder caused by a deficiency or dysfunction of ADAMTS13 and can occur secondary to various conditions, including autoimmune diseases, infections, medications, pregnancy, and malignancies. Diabetic ketoacidosis (DKA) inducing TTP is uncommon and not widely reported in the literature. Herein, we report a case of TTP induced by DKA in an adult patient. His clinical picture, serological, and biochemical results confirmed the diagnosis of TTP induced by DKA, and his clinical course did not improve despite normalization of glucose level, plasmapheresis, and aggressive management. Our case report emphasizes the importance of considering TTP as a potential complication of DKA.

## Introduction

Diabetic ketoacidosis (DKA) is a well-known complication of diabetes mellitus (DM). It is characterized by hyperglycemia, ketonemia, and metabolic acidosis [1]. Thrombotic thrombocytopenic purpura (TTP) is a rare but potentially life-threatening complication of DKA that is not commonly recognized [2]. TTP is a group of disorders characterized by microvascular thrombosis, hemolytic anemia, and thrombocytopenia. TTP can be caused by a variety of conditions, including infections, medications, malignancies, and autoimmune disorders [3]. In this case report, we present a rare case of TTP that occurred in a patient with DKA.

## Case presentation

A 45-year-old male with a history of type 1 diabetes mellitus (DM) presented to the emergency department with complaints of severe abdominal pain, vomiting, and altered sensorium for the last 24 hours. He reported not taking his insulin dose for the last three days due to financial constraints. On examination, he was confused and lethargic with a blood pressure of 145/85 mmHg, heart rate of 110/minute, and respiratory rate of 20/minute. He had dry oral mucosa, diffuse abdominal tenderness, and Kussmaul breathing. Initial laboratory investigation and arterial blood gas analysis showed hyperglycemia and anion gap metabolic acidosis (Table 1). He was diagnosed with DKA and commenced on intravenous fluids, intravenous insulin, and broad-spectrum antibiotics. He was admitted to the intensive care unit (ICU) for further management.

**Table 1:** Initial laboratory results.

Parameter	Lab value	Reference range
Blood glucose level	459 mg/dl	< 250
Hemoglobin	9 g/dl	12.5-13.5
Red cell count	4.1 million cell/m^3^	4.3-5.6
Platelet count	78,000 /m^3^	150,000-350,000
White cell count	9000/m^3^	4000-11000
Arterial pH	7.1	7.35-7.45
Serum bicarbonate	11 mEq/l	22-26
Serum creatinine	1.9 mg/dl	0.6-1.1
Serum ketone level	3.1 mmol/l	<0.6
Sodium	131 mEq/l	135-146
Potassium	4.1 mEq/l	3.1-5.2
Chloride	96mEq/L	96-112
Lactate dehydrogenase	588 IU/l	140-280

On the second day, he remained confused and lethargic. His laboratory investigations revealed worsening thrombocytopenia with a platelet count of 51,000/mm^3^ and anemia with hemoglobin level of 8.5 g/dl. His creatinine level also increased to 2.2 mg/dl with an elevated lactate dehydrogenase level. Peripheral blood smear showed schistocytes, thrombocytopenia, and fragmented RBCs with reticulocytosis, suggestive of microangiopathic hemolytic anemia (MAHA). Further workup included a direct Coombs test and stool examination for Shiga toxin, autoimmune screening, and viral serology, including human immunodeficiency virus (HIV), hepatitis A and B, and coronavirus disease 2019 (COVID-19), which were negative. He was tested for serum disintegrin and metalloproteinase with thrombospondin subtype 1-13 (ADAMTS13) level, which was 21% (Ref>66%) and elevated titer of ADAMTS13 antibody titer 101 (Ref<12 IU/ml). His clinical picture and serological and biochemical test results were suggestive of TTP induced by DKA in the absence of other etiologies.

**Figure 1:**
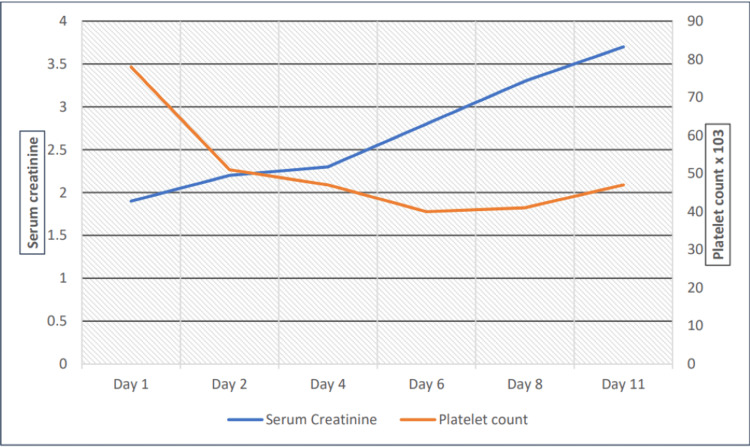
Platelet count and serum creatinine level during hospital stay.

**Figure 2:**
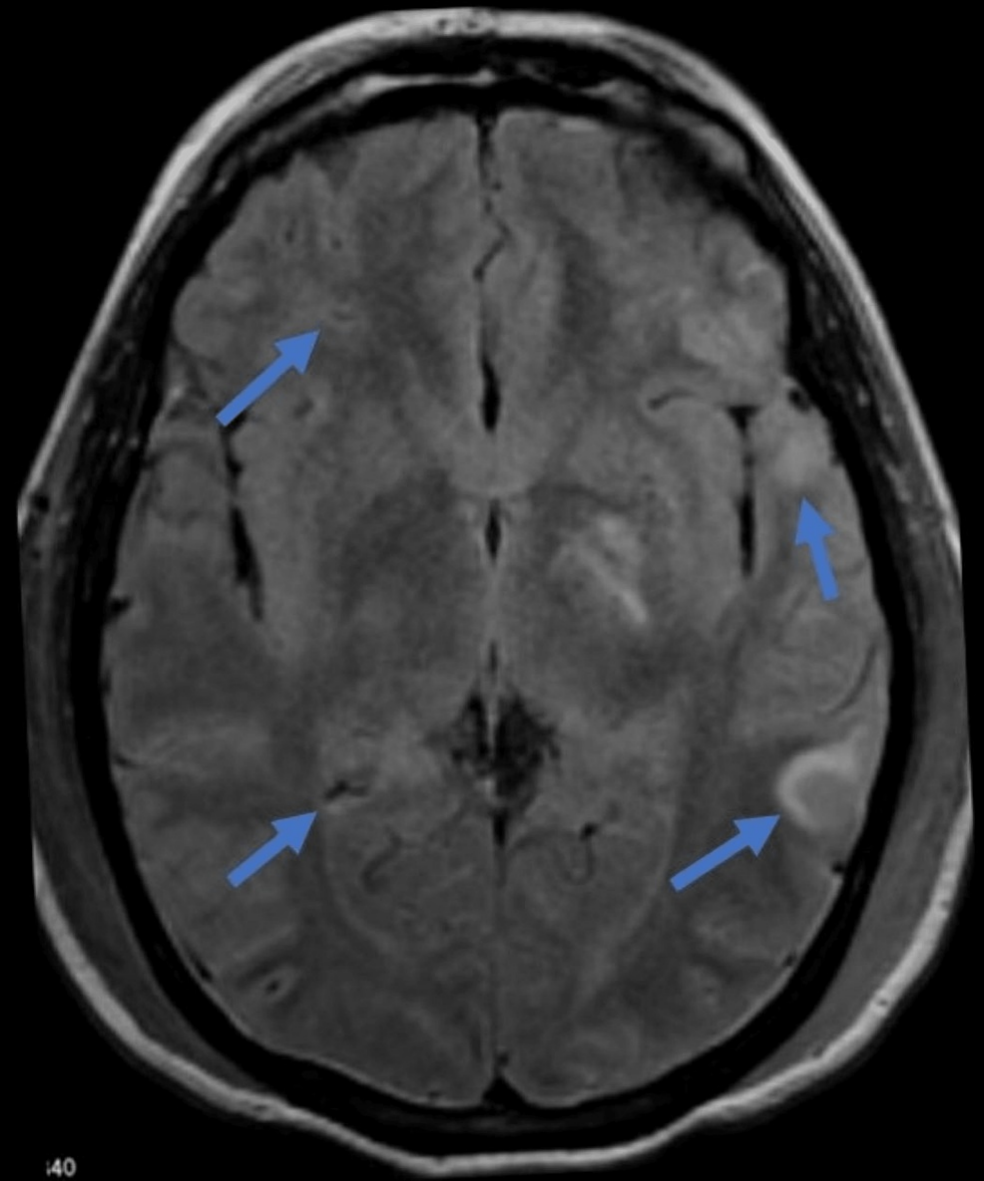
Brain MRI demonstrating diffuse white matter abnormalities and multiple small punctate hemorrhages suggestive of microangiopathy. MRI: magnetic resonance imaging.

He was managed with plasma exchange therapy, intravenous methylprednisolone 1 mg/kg daily, followed by weekly rituximab. He was continued on insulin, broad-spectrum antibiotics, and supportive care for DKA. His clinical course was complicated by worsening renal function (Figure 1). His platelet count and Hb levels continued to remain low despite plasmapheresis. On day six, he developed seizures, followed by lower limb weakness. Brain magnetic resonance imaging (MRI) revealed diffuse white matter abnormalities and multiple small punctate hemorrhages (Figure 2).

On day nine, his clinical course was complicated by acute respiratory distress with sudden onset dyspnea, hypoxemia, rapid shallow breathing, and diffuse bilateral consolidation with air bronchogram, requiring elective intubation, ventilator support and prone positioning along with broad-spectrum antibiotics. Despite aggressive management, his clinical condition continued to deteriorate, and he eventually expired on day eleven of admission.

## Discussion

TTP is a rare but severe complication of DKA that is characterized by microangiopathic hemolytic anemia, thrombocytopenia, and organ dysfunction. It can occur due to various underlying causes, such as infections, autoimmune diseases, medications, or genetic mutations. The exact incidence of thrombotic microangiopathy (TMA) syndromes in DKA is not well known, as the condition is rare and often underdiagnosed. However, several studies have reported a significant association between DKA and TMA, suggesting that the incidence of TMA in DKA may be higher than previously thought [4]. Jackson et al. described a case of TTP in an old male triggered by new onset DKA [5]. After managing DKA and TTP simultaneously, his condition improved with the resolution of both TTP and DKA. Zhu et al. highlighted a case of a diabetic patient who presented with DKA and typical manifestations of microangiopathy suggestive of TMA [6]. Another case report presented a case of DKA-induced TTP in a young patient. Initially, she presented with signs and symptoms of DKA. However, on the day of admission four, she started having manifestations of TTP, including thrombocytopenia and microangiopathy [4]. Khan et al. underlined two cases of TTP precipitated by diabetic ketoacidosis in two young patients who presented with new onset DKA [7].

The pathophysiology of TTP in patients with DKA is not well understood, but several mechanisms have been proposed. The hyperglycemia and acidosis associated with DKA can lead to endothelial damage and platelet activation, leading to microvascular thrombosis [8]. In addition, insulin deficiency can lead to a decrease in the activity of antithrombotic factors, such as tissue plasminogen activator (tPA), resulting in a prothrombotic state [9]. Generally, the release of pro-inflammatory cytokines, such as interleukin-6 (IL-6), can also contribute to the development of TTP. Another proposed mechanism is the activation of the complement system, which leads to the formation of the membrane attack complex (MAC) on endothelial cells [10]. The MAC can cause direct damage to the endothelial cells and also trigger the release of proinflammatory cytokines, which can further exacerbate the inflammatory response.

The management of TTP associated with DKA includes a multidisciplinary approach involving intensive care unit management, fluid and electrolyte management, glycemic control, and treatment of the underlying TTP [11]. Patients with TTP associated with DKA often require intensive care unit management with close monitoring of vital signs, urine output, and serum electrolytes [12]. Aggressive fluid and electrolyte replacement is essential to correct dehydration and electrolyte imbalances. However, care must be taken to avoid fluid overload, which can exacerbate TTP. Prompt correction of hyperglycemia is essential in the management of DKA and TTP [13]. Insulin therapy is the mainstay of treatment, with a target blood glucose level of 150-200 mg/dL. The treatment of TTP associated with DKA includes plasma exchange, corticosteroids, and rituximab. Plasma exchange removes the circulating immune complexes and replaces them with fresh plasma, while corticosteroids and rituximab suppress the immune response [14].

Our patient was diagnosed and treated on the line of DKA. The following day, his laboratory results showed worsening thrombocytopenia, anemia, and increased creatinine level with an elevated lactate dehydrogenase level. Peripheral blood smear showed schistocytes, thrombocytopenia, and fragmented RBCs with reticulocytosis was diagnosed with TTP because of elevated titer of ADAMTS13. Further workup included a direct Coombs test and stool examination for Shiga toxin, which were negative. Autoimmune screening, and viral serology, including human immunodeficiency virus (HIV), hepatitis A and B, and coronavirus disease 2019 (COVID-19), which were negative. His clinical picture and serological and biochemical test results were suggestive of TTP induced by DKA in the absence of other etiologies. The prognosis of TTP associated with DKA is variable and depends on the severity of the underlying condition, the promptness of diagnosis and treatment, and the presence of any comorbidities. Without prompt and effective management, TTP can lead to multiorgan failure, including kidney failure, neurologic deficits, and even death [15].

## Conclusions

Our case report highlights the potential association between DKA and TTP, which is life-threatening. TTP is a rare but serious complication of DKA that requires prompt recognition and management to prevent multiorgan dysfunction and improve patient outcomes. Early and aggressive management with a multidisciplinary approach can help improve the prognosis of this condition.

## References

[1] Joly BS, Coppo P, Veyradier A (2017). Thrombotic thrombocytopenic purpura. Blood.

[2] Saha M, McDaniel JK, Zheng XL (2017). Thrombotic thrombocytopenic purpura: pathogenesis, diagnosis and potential novel therapeutics. J Thromb Haemost.

[3] Sravanthi MV, Suma Kumaran S, Sharma N, Bojanapally P (2020). A rare case of acquired thrombotic thrombocytopenic purpura triggered by acute pancreatitis. Cureus.

[4] Alsaied T, Goldstein SL, Kaddourah A, Poynter SE (2016). Thrombocytopenia-associated multi-organ failure caused by diabetic ketoacidosis. Pediatr Int.

[5] Jackson LJ, Fischer H, Abdelsayed N, Carter M (2021). Diabetic ketoacidosis: possible cause of thrombotic thrombocytopenic purpura. Cureus.

[6] Zhu Z, Chen H, Gill R, Wang J, Spitalewitz S, Gotlieb V (2016). Diabetic ketoacidosis presenting with atypical hemolytic uremic syndrome associated with a variant of complement factor B in an adult: a case report. J Med Case Rep.

[7] Khan MR, Maheshwari PK, Haque A (2013). Thrombotic microangiopathic syndrome: a novel complication of diabetic ketoacidosis. Indian Pediatr.

[8] Reese JA, Muthurajah DS, Kremer Hovinga JA, Vesely SK, Terrell DR, George JN (2013). Children and adults with thrombotic thrombocytopenic purpura associated with severe, acquired Adamts13 deficiency: comparison of incidence, demographic and clinical features. Pediatr Blood Cancer.

[9] Hassan A, Iqbal M, George JN (2019). Additional autoimmune disorders in patients with acquired autoimmune thrombotic thrombocytopenic purpura. Am J Hematol.

[10] Abbas Q, Arbab S, Haque AU, Humayun KN (2018). Spectrum of complications of severe DKA in children in pediatric Intensive Care Unit. Pak J Med Sci.

[11] Scully M, Hunt BJ, Benjamin S (2012). Guidelines on the diagnosis and management of thrombotic thrombocytopenic purpura and other thrombotic microangiopathies. Br J Haematol.

[12] Hermelin D, Blackall D (2020). Successful plasma exchange in a 34-year-old woman with diabetic ketoacidosis and a thrombotic microangiopathy. J Clin Apher.

[13] Little DJ, Reese JA, Vesely SK, George JN (2014). Increased urinary albumin excretion following recovery from thrombotic thrombocytopenic purpura due to acquired ADAMTS13 deficiency. Am J Kidney Dis.

[14] Page EE, Kremer Hovinga JA, Terrell DR, Vesely SK, George JN (2017). Thrombotic thrombocytopenic purpura: diagnostic criteria, clinical features, and long-term outcomes from 1995 through 2015. Blood Adv.

[15] Mostofizadeh N, Arefnia S, Hashemipour M, Dehkordi EH (2018). Thrombotic thrombocytopenic purpura in a child with diabetic ketoacidosis. Adv Biomed Res.

